# High-throughput, non-invasive prenatal testing for fetal rhesus D status in RhD-negative women: a systematic review and meta-analysis

**DOI:** 10.1186/s12916-019-1254-4

**Published:** 2019-02-14

**Authors:** Huiqin Yang, Alexis Llewellyn, Ruth Walker, Melissa Harden, Pedro Saramago, Susan Griffin, Mark Simmonds

**Affiliations:** 1 University of Exeter Medical School, St Luke’s Campus, Heavitree Road, Exeter, EX1 2LU UK; 2 Centre for Reviews and Dissemination, University of York, York, YO10 5DD UK; 3 Centre for Health Economics, University of York, York, YO10 5DD UK

**Keywords:** Fetal rhesus D status, Non-invasive prenatal testing, Diagnostic accuracy, Anti-D immunoglobulin, Systematic review

## Abstract

**Background:**

High-throughput non-invasive prenatal testing (NIPT) for fetal Rhesus D (RhD) status could avoid unnecessary treatment with anti-D immunoglobulin for RhD-negative women found to be carrying an RhD-negative fetus. We aimed to assess the diagnostic accuracy of high-throughput NIPT for fetal RhD status in RhD-negative women not known to be sensitized to the RhD antigen, by performing a systematic review and meta-analysis.

**Methods:**

Prospective cohort studies of high-throughput NIPT used to determine fetal RhD status were included. The eligible population were pregnant women who were RhD negative and not known to be sensitized to RhD antigen. The index test was high-throughput, NIPT cell-free fetal DNA tests of maternal plasma used to determine fetal RhD status. The reference standard considered was serologic cord blood testing at birth. Databases including MEDLINE, EMBASE, and Science Citation Index were searched up to February 2016.

Two reviewers independently screened titles and abstracts and assessed full texts identified as potentially relevant. Risk of bias was assessed using QUADAS-2. The bivariate and hierarchical summary receiver-operating characteristic (HSROC) models were fitted to calculate summary estimates of sensitivity, specificity, false positive and false negative rates, and the associated 95% confidence intervals (CIs).

**Results:**

A total of 3921 references records were identified through electronic searches. Eight studies were included in the systematic review. Six studies were judged to be at low risk of bias. The HSROC models demonstrated high diagnostic performance of high-throughput NIPT testing for women tested at or after 11 weeks gestation. In the primary analysis for diagnostic accuracy, women with an inconclusive test result were treated as having tested positive. The false negative rate (incorrectly classed as RhD negative) was 0.34% (95% CI 0.15 to 0.76) and the false positive rate (incorrectly classed as RhD positive) was 3.86% (95% CI 2.54 to 5.82). There was limited evidence for non-white women and multiple pregnancies.

**Conclusions:**

High-throughput NIPT is sufficiently accurate to detect fetal RhD status in RhD-negative women and would considerably reduce unnecessary treatment with routine anti-D immunoglobulin. The applicability of these findings to non-white women and women with multiple pregnancies is uncertain.

**Electronic supplementary material:**

The online version of this article (10.1186/s12916-019-1254-4) contains supplementary material, which is available to authorized users.

## Introduction

Pregnant women who have an RhD-negative blood type may carry an RhD-positive fetus. The presence of fetal RhD-positive cells in the maternal circulation can cause a mother who is RhD negative to produce anti-D antibodies against the RhD antigen. This immune response, termed sensitisation, can happen at any time during the pregnancy, but it is most common in the third trimester and during childbirth [1].

The process of sensitisation itself has no adverse effects on the mother and does not usually affect the pregnancy during which it occurs. However, in a subsequent pregnancy with an RhD-positive fetus in women who have been sensitized to the RhD antigen, the woman’s anti-D antibodies may cross the placenta resulting in haemolytic disease of the fetus and newborn.

This can cause severe fetal anemia that leads to fetal heart failure, fluid retention and swelling (hydrops), hyperbilirubinaemia, kernicterus, and perinatal death [2].

Prophylaxis with anti-RhD immunoglobulin can substantially reduce the risk of sensitisation in RhD-negative women and hence the prevalence of haemolytic disease of the fetus and newborn [3]. The introduction of routine antenatal prophylaxis during the third trimester of pregnancy has led to a reduction in sensitisation, resulting in a decrease in mortality associated with haemolytic disease of the fetus and newborn from 46 in 100,000 births before 1969 to 1.6 in 100,000 births by 1991 [4].

Currently, the National Institute for Health and Clinical Excellence (NICE) guideline on antenatal care recommends that women should be offered testing for blood group and rhesus D status in early pregnancy [5]. In those identified as RhD negative and without preformed antibodies, administration of anti-D immunoglobulin is recommended both as prophylaxis and following potential sensitizing events to prevent the sensitisation from occurring. Routine antenatal prophylaxis with anti-D immunoglobulin can be given as two doses at weeks 28 and 34 of pregnancy or as a single dose between 28 and 30 weeks [5].

Non-invasive prenatal testing (NIPT) of fetal RhD status uses a real-time quantitative polymerase chain reaction (PCR) method to detect cell-free fetal DNA—small fragments of extracellular DNA shed from the placenta circulating freely in the maternal plasma. High-throughput NIPT performs the test using an automated platform capable of performing a large number of tests simultaneously, and is therefore suitable for large-scale population screening of pregnant women. High-throughput NIPT for fetal RhD status may enable anti-D immunoglobulin to be withheld from RhD-negative women who are predicted to be carrying an RhD-negative fetus. Pregnant women found to be carrying an RhD-negative fetus could avoid unnecessary treatment with anti-D immunoglobulin (which is a human polyclonal plasma-derived product), along with the potential risk associated with administration of blood products. In addition, these women may not need the provision of anti-D immunoglobulin following potentially sensitizing events, and there may no longer be a need for serologic cord testing at birth. High-throughput NIPT is already used in this way in some European countries [6, 7].

However, the diagnostic accuracy of high-throughput NIPT for fetal Rhesus D status in RhD-negative women not known to be sensitized to the RhD antigen is uncertain. The National Institute of Health Research Health Technology Assessment programme commissioned a systematic review and economic evaluation to assess the diagnostic accuracy and cost-effectiveness of high-throughput NIPT for the detection of fetal Rhesus D status in RhD-negative women. This systematic review formed part of a larger report on high-throughput NIPT which also considered broader issues in its clinical value and implementation and a full economic analysis [8]. This work was used to inform the recent NICE guideline regarding the recommendation of high-throughput NIPT for fetal Rhesus D status [9].

## Methods

The complete methodology of the original wider review is reported elsewhere [8]. This section summarizes the methodology of the diagnostic accuracy review.

### Data sources and searches

We conducted a systematic review following the PRISMA statement [10] and registered the review on PROSPERO. The following databases were searched for relevant studies from inception to November 2015: MEDLINE, MEDLINE In-Process, CINAHL, Cochrane Central Register of Controlled Trials (CENTRAL), Cochrane Database of Systematic Reviews (CDSR), Database of Abstracts of Reviews of Effects (DARE), EMBASE, Health Technology Assessment (HTA) database, Maternity and Infant Care, PubMed, and the Science Citation Index. In addition, the following resources were searched for on-going, unpublished, or gray literature: ClinicalTrials.gov, Conference Proceedings Citation Index: Science, EU Clinical Trials Register, PROSPERO, and the WHO International Clinical Trials Registry Platform portal. An updated search was performed in February 2016.

Both published and unpublished literature were identified from systematic searches of electronic sources, consultation with experts in the field, and reference checking of relevant systematic reviews and included studies. Search strategies were developed by an information specialist (MH). The search strategy can be found in Additional file 1.

### Study selection

Prospective cohort studies of high-throughput NIPT used to determine fetal RhD status were eligible for inclusion. We considered as high-throughput, any NIPT tests which were conducted using an automated robotic platform (including automated DNA extraction and liquid handling) and were able to process large numbers of samples rapidly for large-scale screening purposes. Studies where this test was used for diagnosis (rather than screening) of sensitized women were excluded.

The inclusion criteria for population, index test, reference standard, and outcomes are listed below:

- The eligible population were pregnant women who were RhD negative and not known to be sensitized to RhD antigen.
- The index test was high-throughput, NIPT cell-free fetal DNA tests of maternal plasma used to determine fetal RhD status.
- The reference standard considered was serologic cord blood testing at birth.
- The eligible studies had to report diagnostic accuracy data such as absolute numbers of true positive, false positive, true negative, and false negative test results.

Two researchers independently screened the titles and abstracts of all reports identified by the search strategy, and full-text papers were subsequently obtained for assessment. Only reports published in English were sought. There were no restrictions for publication date. Full-text copies of all studies deemed to be potentially relevant were obtained and two reviewers independently assessed them for inclusion (HY, AL). Any disagreements were resolved by consensus or by a third reviewer (RW).

### Data extraction

We selected the most recent or most complete report in cases of multiple reports for a given study or when we could not exclude the possibility of overlapping populations. One reviewer independently extracted details from full-text studies including study design, participants, index, comparator and reference standard tests, and outcome data. The data extraction was checked by another reviewer. Any disagreements were resolved by consensus (between HY and AL) or with a third reviewer (RW).

We extracted the number of true positives, true negatives, false positives, and false negatives for each index test evaluated in each study in order to construct 2 × 2 tables. If reported, we extracted data on the number of undetermined or uninterpretable results. Study authors were contacted if some data were unclear or missing.

### Quality assessment

Risk of bias was assessed using a modified version of the quality assessment of diagnostic accuracy studies (QUADAS-2) checklist [11]. The QUADAS-2 tool consists of four key domains: (1) patient selection, (2) index test, (3) reference standard, and (4) flow of patients through the study and timing of the index test(s) and reference standard. Each domain was assessed in terms of the risk of bias. The first three domains were also assessed for concerns regarding their applicability in terms of whether (1) the participants and setting, (2) the index test, its conduct or interpretation, and (3) the target condition as defined by the reference standard were applicable to the UK context. One reviewer (AL) independently assessed the quality of all included studies in terms of risk of bias. The quality assessment was checked by another reviewer (HY). Any disagreements were resolved by consensus or by a third party (RW).

### Data synthesis

For diagnostic accuracy outcomes, estimates of sensitivity, specificity, and false positive and false negative rates were calculated and presented on forest plots and in receiver-operating characteristic (ROC) space to assess the heterogeneity in test accuracy within and between studies. The hierarchical bivariate model [12] was fitted to calculate summary estimates of sensitivity, specificity, and false positive and false negative rates and the associated 95% confidence intervals (CIs). The hierarchical summary ROC (HSROC) model [13] was fitted to produce summary ROC curves. Both models jointly model sensitivity and specificity and account for the correlation between them. Heterogeneity in sensitivity and specificity was also assessed using the *I*^2^ statistic. All analyses were performed using R software [14, 15]. Because NIPT testing is highly accurate, we present the results in terms of the false positive rate (FPR) (incorrectly testing positive and being offered unnecessary anti-D prophylaxis) and false negative rate (FNR) (incorrectly testing negative; at risk of sensitisation as women do not receive anti-D prophylaxis), rather than the conventional sensitivity and specificity.

Some NIPT results are inconclusive and unable to predict the RhD status of the fetus. Current UK practice is to treat such test results as if they predicted an RhD-positive fetus, and this approach was used in the primary analysis of diagnostic accuracy. Sensitivity analyses were conducted to explore the robustness of the results by including and excluding such inconclusive test results. A further sensitivity analysis included only UK (Bristol)-based studies, as this review was intended to inform UK practice. Furthermore, as test accuracy may vary according to the gestation age when NIPT is performed, we investigated the impact of test timing by plotting diagnostic accuracy against time, and performing meta-regressions against test timing. No analysis for small study effects or publication bias was performed because there were too few studies identified to justify this.

## Results

**Fig. 1:**
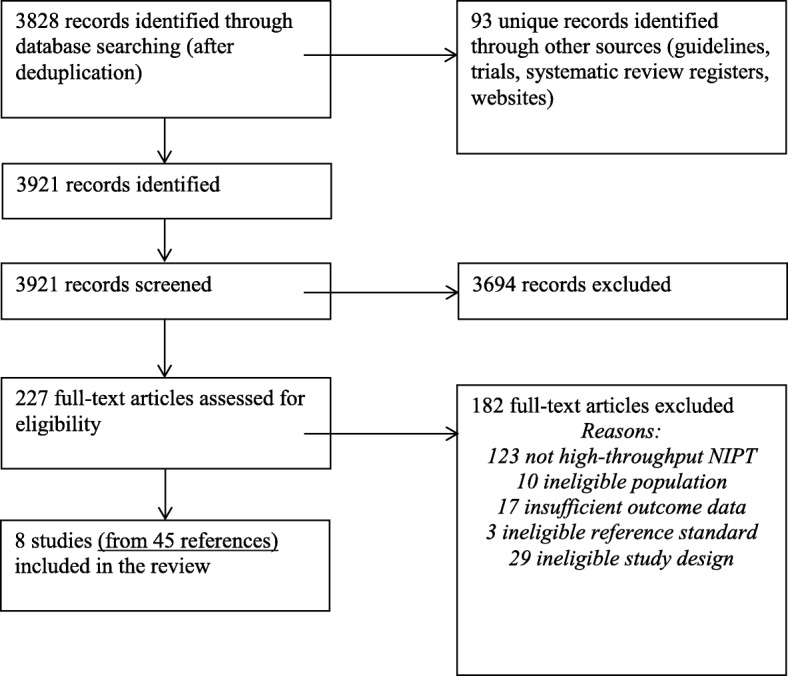
Flow diagram: study selection process

The literature searches of bibliographic databases identified 3921 references. After initial screening of titles and abstracts, 227 were considered to be potentially relevant and were ordered for full paper screening. In total, eight studies [6, 7, 16–19] were included in the diagnostic review of high-throughput NIPT testing. Six studies reported inconclusive results. Figure 1 shows a flow diagram outlining the screening process with reasons for exclusion of full-text papers.

Table 1 presents the summary information of characteristics of the included diagnostic accuracy studies. All the studies were prospective studies and conducted in European countries. Four studies were conducted in England [16–19], three of which were based at Bristol [16–18]. The sample size of studies ranged from 282 to 18,383. Most studies recruited pregnant women with a gestational age of 10 to 28 weeks. Most participants were white, and most pregnancies were singleton. All studies used maternal plasma as their sample source. A robotic DNA extraction instrument was employed in all studies. The studies used a number of robotic platforms including MDx BioRobot, MagNa Pure 96, MagNA Pure LC, and COBAS AmpliPrep. For PCR, all studies targeted at least two exons (generally exons 5 and 7) and at least two controls for *RHD* assay (RhD-positive DNA and RhD-negative DNA) except for the study by Wikman et al. [20] which targeted exon 4 only and used *GAPDH* DNA as control. The reference standard used in all studies was cord blood serology, except for Akolekar et al. [17] which did not describe its reference standard. Where reported, rates of inconclusive results ranged from 1.0% [20] to 14.3% [19].

**Table 1:** Characteristics of the diagnostic accuracy studies

Study (First author/year)	Location	DNA extraction tool	Exons targeted	Controls	Reference standard	Gestational age at time of NIPT (median/range)	Sample size^α^	Confirmed RhD positive	Confirmed RhD negative	Inconclusive test results (%)	Reasons for inconclusive results (*n* of cases)
Akolekar 2011 [19]	UK (London)	MDx BioRobot (Qiagen)	5 and 7	RhD+, RhD–, RHDΨ +, No DNA	“Serologically at delivery”	12.4 (11–14)	586	410	176	84 (14.3)	Maternal or fetal RHD variant and insufficient maternal plasma for further analysis (NR)^!^
Banch Clausen 2014 [6]	Denmark	QIAsymphony SP; MagNA Pure LC; MagNA Pure Compact Instrument (Roche)	5 and 10, or 5 and 7, or 7 and 10	RhD+, RhD–^£^	Cord blood serology^~^	25 (23–28)	12,668	7830	4838	274 (2.2)	Maternal weak D (93); maternal silent RHD variant (38); high level of maternal background DNA (29); technical problems (19); maternal D^VI^ (14); weak PCR signal (13); suspected maternal RHD positive (3); no reported cause (65)
Chitty 2014 [16]	UK (Bristol)	MDx BioRobot (Qiagen)	5 and 7	RhD+, RhD–, RHDΨ+, No DNA	Cord blood serology	19 (5–35)	4913	2890	2023	393 (8.0)	NR
Finning 2008 [17]	UK (Bristol)	MDx BioRobot (Qiagen)	5 and 7	RhD+, RhD–, RHDΨ+, No DNA	Cord blood serology^>^	28 (8–38)	1869	1156	713	56 (3.0)	Insufficient DNA (30); suspected maternal RHD gene (25); failure to extract DNA from plasma (1)
Grande 2013 [25]	Spain	COBAS AmpliPrep (Roche)	5 and 7, 10^$^	RhD+, RhD–	Cord blood serology	24–26	282	186	96	NR	NR
Soothill 2015 [18]	UK (Bristol)	MDx BioRobot (Qiagen)	5 and 7	RhD+, RhD–, RHDΨ+, No DNA	Cord blood serology	15–17 (mostly)	499*	315	184	61 (12.2)	NR
Thurik 2015 [7]	Netherlands	MagNa Pure 96 (Roche)	5 and 7	RhD+, RhD–	Cord blood serology^^^	26	18383*	11,283	7100	NR	NR
Wikman 2012 [20]	Sweden	MagNA Pure LC (Roche)	4	GAPDH	Serology from cord blood or citrate samples from newborns^+^	8–40	3291^#^	2073	1218	32 (1.0)	RHD variant (14); no second sample (18, of which 13 were spontaneous abortions and miscarriages)

^α^Number of blood samples unless otherwise specified; * number of participants; ^#^ excludes pre-8 weeks gestation pregnancies; ^$^ on 2nd DNA extraction, only to confirm RHD-negative results; ^£^ Multiple controls without template were also included using sterile H_2_O; ^!^ 5 mothers with insufficient DNA were excluded from the analyses and not classed as inconclusive

NR: not reported; RhD+: RhD positive; RhD−: RhD negative; PCR: Polymerase chain reaction; GAPDH: glyceraldehyde-3-phosphate dehydrogenase; ^~^ Region 1: ID-Card DiaClon ABD-Confirmation for donors, (DiaMed GmbH 1785 Cressier FR Switzerland) with monoclonal anti-D (cell lines ESD-1M, 175–2) that detects most weak RhD types and partial D^VI^ phenotype; Region 2: direct agglutination in a gel matrix test with IgM monoclonal anti-D clone 175–2 (DiaMed); further tests in gel matrix test with in-house Dw1 anti-D for initial negative tests. For discrepancies, DNA extracted from the cord blood and tested for RHD exon 10 and further analyzed by PCR-SSP using the RH-type kit (Biologische Analysensystem GmbH, Lich, Germany); Region 3: Direct agglutination with monoclonal antibody Diagast anti-D IgM (ref. no. 71000) for RHD positive. For unexpectedly negative reactions, an additional IAT with anti-D IgG LOR17 was performed. IAT with anti-D IgG LOR17 was used for RHD negative; Region 4: Serological testing of cord blood RBC was done by using Seraclone Anti-D (RH1) Blend; Ref 802032 (Biotest, Germany); Region 5: Serological testing of cord blood RBC with 2 complete anti-Ds (Medion Diagnostics, IgM anti-D[MS201] and Seraclone, [Rh1] 226) in saline. For reactions of less than 3+ for both reagents, further investigation for D expression by IAT with two IgG anti-Ds. ^>^ No DNA extraction of cord blood samples. ^^^ Cord blood serology with WA-Diana system (DiaMed GmbH) using two monoclonal anti-D reagents, LHM 59/20 (LDM3) + 175–2 and ESD- 1M+ 175–2. Maternal and fetal RHD variant genes were analyzed with an RHD-multiplex ligation-dependent probe amplification (MLPA) assay on genomic DNA. Mutation analysis and copy number variation investigated via RHD MLPA. Discordant positive results due to maternal or fetal RHD variants were identified and excluded from the study. Samples with weak PCR signals were excluded. The Kleihauer–Betke test and multiplex short tandem repeat (STR)-PCRs on 15 systems on leukocyte-derived DNA were used exclude errors around cord blood collection. ^+^ Blood typing using DiaClon ABO/Rh for Newborns DVI+ gelcards

### Risk of bias

Table 2 presents a summary of the results for the risk of bias assessment. The majority of included studies were judged to be at low risk of bias, but two studies [7, 19] were judged to be at high risk of bias. The study by Akolekar et al. [19] reported that the targeted RhD-negative women were selected from a database, but it was unclear whether this selection was conducted on a random basis. The study enrolled a large proportion of Africans (19.3%) which may not be representative of the general population of pregnant women in the UK. This may have contributed to the larger than average proportion of inconclusive results (14.3%). Characteristics of the reference standard were also poorly reported in this study. In the study by Thurik et al. [7], only 80% of participants received a reference standard. The reasons why cord blood serology was not conducted in a significant proportion of the study population were not stated. This study also reported that its prediction algorithm was judged daily and modified as needed, which may have introduced bias in the diagnostic accuracy estimates.

**Table 2:** Risk of bias of included studies

Study	Risk of bias				Applicability concerns		
	Patient selection	Index test	Reference standard	Flow and timing	Patient selection	Index test	Reference standard
Akolekar (2011) [19]	High	High	Unclear	Unclear	High	Low	Unclear
Banch-Clausen (2014) [6]	Low	Low	Low	Low	Unclear	Low	Low
Chitty (2014) [16]	Low	Low	Low	Low	Low	Low	Low
Finning (2008) [17]	Low	Low	Low	Low	Low	Low	Low
Grande (2013) [25]	Low	Low	Low	Low	Low	Low	Low
Soothill (2015) [18]	Low	Unclear	Low	Low	Low	Low	Low
Thurik (2015) [7]	Low	High	Low	High	Low	Low	Low
Wikman (2012) [20]	Low	Low	Low	Low	Unclear	High	Low

High: high risk of bias; Low: low risk of bias

NIPT as an automated procedure was deemed to be of limited risk to human error, and multiple controls were used for *RHD* assays in all except one study [20]. The index test of NIPT was conducted independent of the reference standard, and the results of one were considered unlikely to influence the results of the other; therefore, the risk of incorporation bias was considered low. It appears that most studies prospectively recruited consecutive samples from clinical practice. Only three studies stated that their diagnostic threshold was pre-specified during the conduct of the screening program [6, 16, 17].

The results of the studies were considered broadly applicable to the use of high-throughput NIPT for nationwide screening purposes, except for two studies [19, 20]. In particular, the NIPT test used in the study by Wikman et al. [21] only targeted exon 4, unlike all other included studies where at least two exons (5, 7, and/or 10) were targeted. It is generally accepted that a combination such as of exons 5 and 7 should be targeted to discriminate the pseudogene *RHD*Ψ, particularly present in individuals of African origin [22]. 

### Meta-analysis

The results of the bivariate meta-analyses are shown in Table 3. These show that NIPT is a highly accurate test. The false negative rate (where women would not be offered anti-D prophylaxis and so be at risk of sensitisation) is very low at 0.34% (95% CI 0.15 to 0.76). When treating women with an inconclusive test result as if they were positive, the false positive rate is 3.86% (95% CI 2.54 to 5.82). Excluding inconclusive test results reduces this to 1.26% (95% CI 0.87 to 1.83). Therefore, most false positive results occur in women with inconclusive test results.

**Table 3:** Bivariate meta-analyses of false positive and negative rates

Analysis case	Number of studies	False negative rate (at risk of sensitisation)		False positive rate (unnecessary anti-D)	
		Estimate (%)	95% CI	Estimate (%)	95% CI
Inconclusive tests treated as test positive	8	0.34	0.15–0.76	3.86	2.54–5.82
Excluding all women with inconclusive test results	8	0.35	0.15–0.82	1.26	0.87–1.83
Studies conducted in Bristol only	3	0.21	0.09–0.48	5.73	4.58–7.16

There was some evidence of inconsistency across studies. *I*^2^ was 75% for the false negative rate and 99% for the false positive rate. It should be noted that these high heterogeneities are, in part, a consequence of the high accuracy of the test and the large size of the studies (and consequent small within-study variance, because *I*^2^ increases as the average within-study variance declines). They do not necessarily indicate any clinically meaningful differences between studies. The heterogeneity in false positive rates is likely to be a consequence of differing reporting and handling of inconclusive tests.

Studies conducted in Bristol had a lower false negative rate (0.21%, 95% CI 0.09 to 0.48), with a consequently higher false positive rate (5.73%, 95% CI 4.58 to 7.16). This suggests that the Bristol high-throughput NIPT testing approach, in which the MDx Bio Robot machine is used, may be using a different test threshold to other countries, which further minimizes false negative findings.

**Fig. 2:**
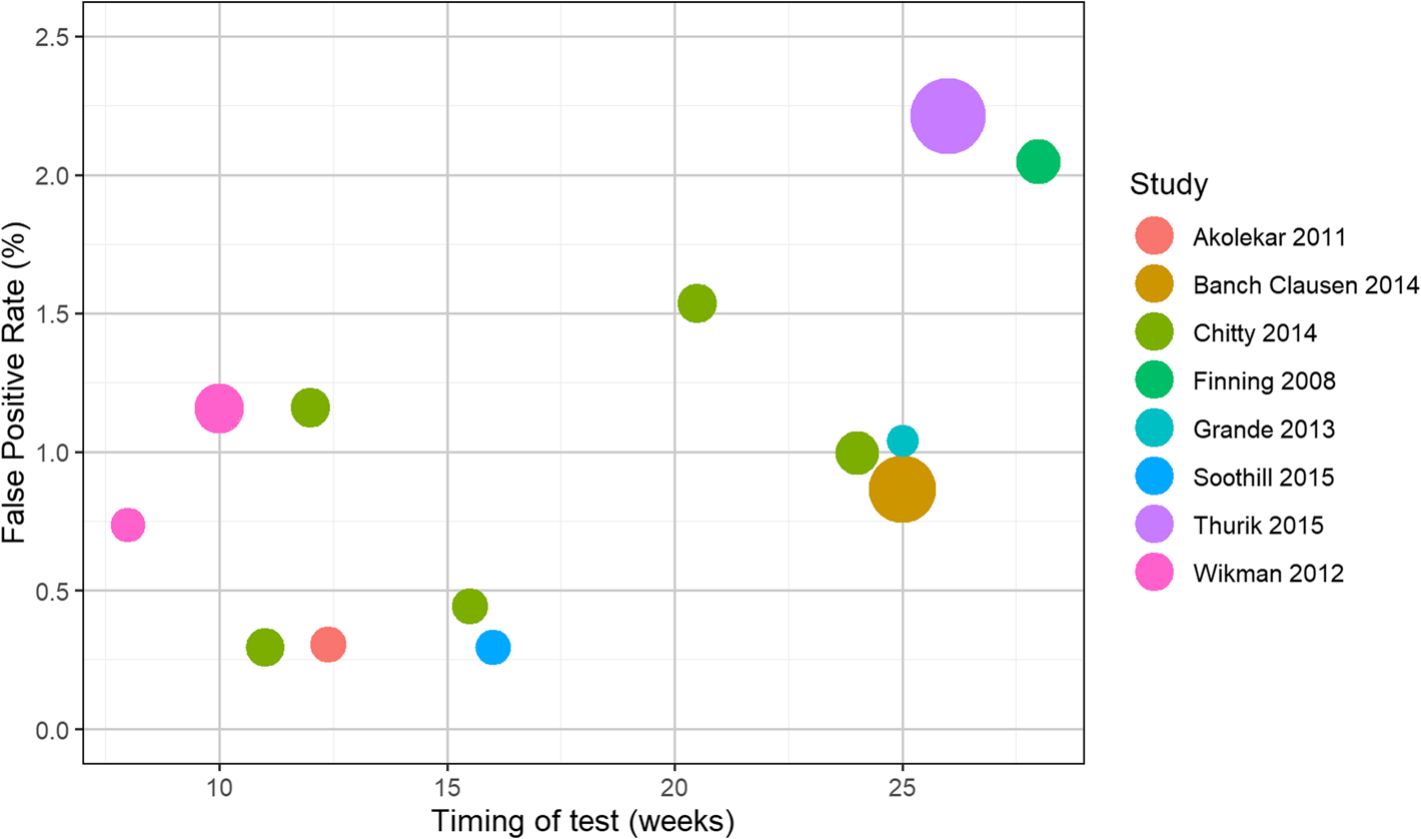
HSROC and bivariate meta-analysis

Figure 2 shows the results of the bivariate and the summary HSROC curve for this primary analysis, which is presented in terms of false positive and false negative rates. The black circle is the summary effect estimate from a bivariate model, and the black curve is the HSROC curve. This plot shows that the studies were generally consistent in terms of false negative results, except for two outlying studies [19, 20]. The study by Wikman et al. [20] conducted most NIPT tests in the first trimester, earlier than other studies. The studies are less consistent in false positive rates. This is most likely because the studies have different numbers of inconclusive test results, and different methods of handling such results.

### Timing of NIPT tests

**Fig. 3:**
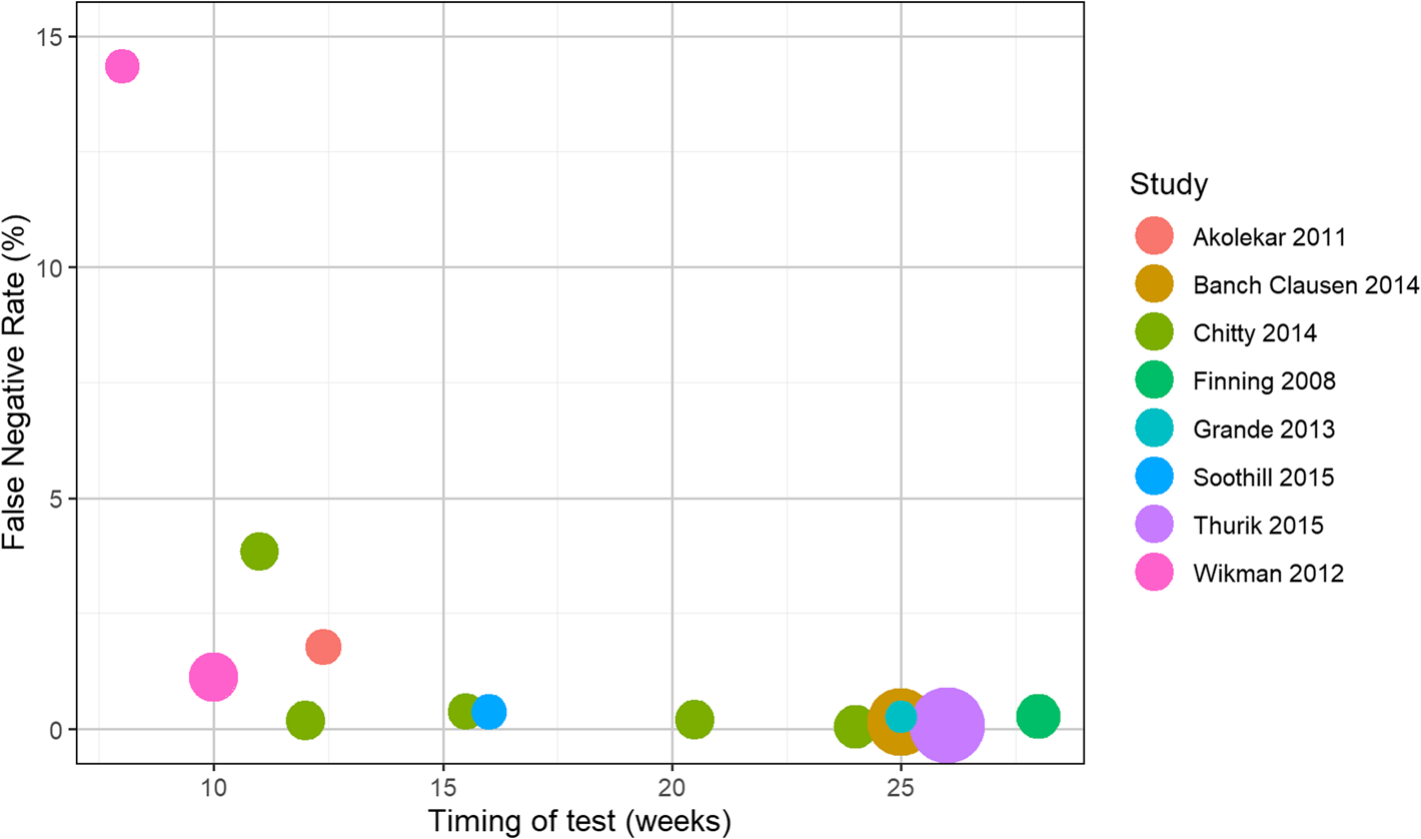
False negative rate by gestational age at time of NIPT

Figure 3 shows the false negative rates plotted by gestational age at time of high-throughput NIPT testing. It suggests that false negative rates after the first trimester (i.e., after around 13 weeks’ gestation) were consistent, irrespective of timing, but false negative rates were higher in the first trimester. This pattern is most visible in the Chitty study [16] which reported diagnostic accuracy at a range of test timings. Given the limited amount of data, no formal statistical test could be performed to confirm this conclusion. Additional file 2: Figure S1 shows the false positive rates plotted by gestational age at time of high-throughput NIPT testing. There was no obvious pattern from this figure.

We also evaluated the impact of the timing of high-throughput NIPT testing on the number of inconclusive test results. As seen in Additional file 3: Figure S2, there is a suggestion that the percentage of inconclusive results for this test reduces as the gestational age increases. This is most obvious in the study by Chitty et al. [16].

### Impact on UK practice

We considered the likely impact of implementing NIPT to guide anti-D prophylaxis in the UK by conducting a simulation study, simulating a representation of the UK population using data sourced from the review (see Additional file 4 for input parameters).

Based on the results of the three Bristol-based studies, we assumed that 60.7% of RhD-negative women have an RhD-positive fetus, and 6.7% of women have an inconclusive NIPT result. Given this, the results of the diagnostic meta-analysis, and parameters described in Additional file 4, using NIPT would result in anti-D prophylaxis being received by 65.9% of RhD-negative women. It would reduce the numbers of women receiving unnecessary anti-D from 38.9 to 5.7%. The number of women who miss out on potentially beneficial anti-D would rise from 0.6 to 1.2%, leading to potentially more sensitisations: an extra 3 per 100,000 women if postpartum cord blood testing continues, or 13 per 100,000 if it is withdrawn. Sensitisation rates using universal anti-D administration were estimated to be 280 per 100,000 women, so this increase is small.

It would however mean 0.12% of women with an RhD-positive fetus would not be offered anti-D and so would be at risk of sensitisation.

## Discussion

In this systematic review, we identified eight studies that evaluated diagnostic accuracy of high-throughput NIPT. Six of these studies were judged to be at low risk of bias. Meta-analyses showed very high diagnostic accuracy of high-throughput NIPT testing.

Diagnostic accuracy of high-throughput NIPT varied by gestational age. The data suggest that high-throughput NIPT testing is insufficiently accurate in the first trimester, but is consistently accurate at any time thereafter. This might be due to low concentration of cell-free fetal DNA in early pregnancy [23] but an increased concentration of cell-free fetal DNA after the end of the first trimester [24].

Given the very high diagnostic accuracy performance of high-throughput NIPT testing, implementing high-throughput NIPT for fetal RhD screening in all RhD-negative women nationwide could be feasible. The results suggest it would substantially reduce the need for antenatal anti-D prophylaxis, while only marginally increasing the risk of sensitisation due to false negative test results. NIPT testing could be conducted, with low false positive rates, at any time from the second trimester onwards, perhaps to coincide with routine antenatal blood tests. Any nationwide NIPT screening program will require careful logistical management to ensure that blood samples are transported to laboratories and tested quickly and that results are reliably returned to general practitioners and midwives.

### Limitations

We performed extensive literature searches with an attempt to maximize retrieval of potentially relevant studies. These included electronic searches of a variety of bibliographic databases as well as screening of clinical trial registers and conference proceedings to identify unpublished studies. However, only studies in English were included; therefore, some potentially relevant non-English language studies may have been missed. There was some evidence of inconsistency in the meta-analysis of diagnostic accuracy studies. The observed heterogeneity may be due to variations in methods used in the high-throughput NIPT approach (e.g., different diagnostic accuracy thresholds used, and different number and types of exons targeted, gestational age at the time of testing, and different methods of handling inconclusive test results). In addition, there was variation in the reporting of included studies. Particularly, two studies [7, 25] did not report the number of inconclusive results of the test and some studies did not report detailed reasons for inconclusive results. The simulation study assumes that the input probabilities are accurate and does not account for any uncertainty in their estimation. Therefore, results of the simulation study should be considered to be illustrative only and not definitive estimates of effect.

### Implications for future research

Further large prospective cohort studies evaluating diagnostic accuracy of high-throughput NIPT in women of non-white ethnicity are required. This is of particular concern as non-white women are more likely to have less accurate test results. For example, in people with African ethnicity, because of the presence of *RHD* pseudogene [26], prenatal detection of fetal RhD type from maternal blood would lead to higher rates of false positive results in this particular population. Further research to improve the NIPT test itself is also warranted, especially for reducing the number of inconclusive test results.

## Conclusions

The findings from this systematic review have demonstrated high diagnostic performance of high-throughput NIPT testing for the detection of fetal RhD status in RhD-negative women, with very low false positive and false negative rates in women tested at or after 11 weeks’ gestation. The use of high-throughput NIPT testing as a routine screening test for fetal RhD status in RhD-negative women can largely remove unnecessary exposure to prophylactic anti-D treatment. Due to limited evidence, the accuracy of NIPT in non-white women and multiple pregnancies is unclear.

## Additional files

Additional file 1: Search strategy. (DOCX 15 kb)

Additional file 2: **Figure S1.** False positive rate by gestational age at time of NIPT. (TIFF 241 kb)

Additional file 3: **Figure S2.** Inconclusive results by test timing. (TIFF 67 kb)

Additional file 4: Probability estimates derived from published data, used in the simulation study. (DOCX 15 kb)
