# Supplementary material for: High-throughput, non-invasive prenatal testing for fetal rhesus D status in RhD-negative women: a systematic review and meta-analysis
Source: BMC Med. 2019 Feb 14;17:37. doi: 10.1186/s12916-019-1254-4 (PMC6375191; doi:10.1186/s12916-019-1254-4)
Supplement: Supplementary file 1 — Search strategy. (DOCX 15 kb) [file 12916_2019_1254_MOESM1_ESM.docx]

**Additional file 1:** **Search strategy**

**MEDLINE**

1 Rh-Hr Blood-Group System/ (10006)

2 (RhD or "rhesus D" or "Rh(D)" or "Rh-(D)" or Rh D).ti,ab. (3323)

3 (Rh-negative or Rh-positive).ti,ab. (898)

4 (Rhesus negative or Rhesus positive).ti,ab. (228)

5 ((rh or rhesus) adj2 (factor or factors or antigen$ or system or group)).ti,ab. (3438)

6 or/1-5 (13812)

7 Rh Isoimmunization/ (1505)

8 ((isoimmuni$ or iso-immuni$ or isoimmune or iso-immune) adj6 (rh or rhesus or maternal or pregnan$)).ti,ab. (1164)

9 ((alloimmuni$ or allo-immuni$ or alloimmune or allo-immune) adj6 (rh or rhesus or maternal or pregnan$)).ti,ab. (870)

10 ((unsensiti#ed or un-sensiti#ed or non-sensiti#ed) adj6 (rh or rhesus or maternal or pregnan$)).ti,ab. (25)

11 ((sensiti#ation$ or sensiti#ed) adj6 (rh or rhesus or maternal or pregnan$)).ti,ab. (1074)

12 ((fetomaternal or feto-maternal or foetomaternal or foeto-maternal) adj2 immuni#ation).ti,ab. (80)

13 ((rh or rhesus) adj2 (immuni#ation or autoimmuni#ation)).ti,ab. (695)

14 or/7-13 (4428)

15 exp Erythroblastosis, Fetal/ (11006)

16 ((hemolytic or haemolytic) adj2 (disease$ or disorder$)).ti,ab. (4465)

17 HDFN.ti,ab. (95)

18 ((rhesus or rh) adj2 (disease$ or disorder$)).ti,ab. (742)

19 ((rhesus or rh or RhD) adj2 (incompatib$ or antagonism)).ti,ab. (750)

20 ((erythroblastoses or erythroblastosis) adj2 f?etal$).ti,ab. (760)

21 or/15-20 (13551)

22 6 or 14 or 21 (25723)

23 Prenatal Diagnosis/ (33273)

24 Maternal Serum Screening Tests/ (153)

25 Hematologic Tests/ (5564)

26 ((prenatal or pre-natal or antenatal or ante-natal) adj3 (test$ or screen$ or diagnos$ or determin$ or detect$)).ti,ab. (32925)

27 ((fetal or foetal or fetus$ or foetus$) adj3 (test$ or screen$ or diagnos$ or determin$ or detect$)).ti,ab. (20036)

28 (NIPD or NIPT).ti,ab. (328)

29 or/23-28 (69981)

30 Genotyping Techniques/ (2761)

31 ((genotype$ or genotyping) adj2 (fetal or foetal or fetus$ or foetus$ or prenatal or pre-natal or antenatal or ante-natal)).ti,ab. (606)

32 ((genotype$ or genotyping) adj2 (maternal or pregnan$)).ti,ab. (789)

33 ((genotype$ or genotyping) adj2 (noninvasive or non-invasive)).ti,ab. (71)

34 cell-free f?etal DNA.ti,ab. (489)

35 cffDNA.ti,ab. (87)

36 or/30-35 (4483)

37 22 and 29 (1795)

38 22 and 36 (276)

39 37 or 38 (1869)

40 (editorial or comment).pt. (946538)

41 39 not 40 (1824)

42 exp animals/ not humans/ (4137930)

43 41 not 42 (1815)
