# Supplementary figures and images for: High-throughput, non-invasive prenatal testing for fetal rhesus D status in RhD-negative women: a systematic review and meta-analysis
Source: BMC Med. 2019 Feb 14;17:37. doi: 10.1186/s12916-019-1254-4 (PMC6375191; doi:10.1186/s12916-019-1254-4)

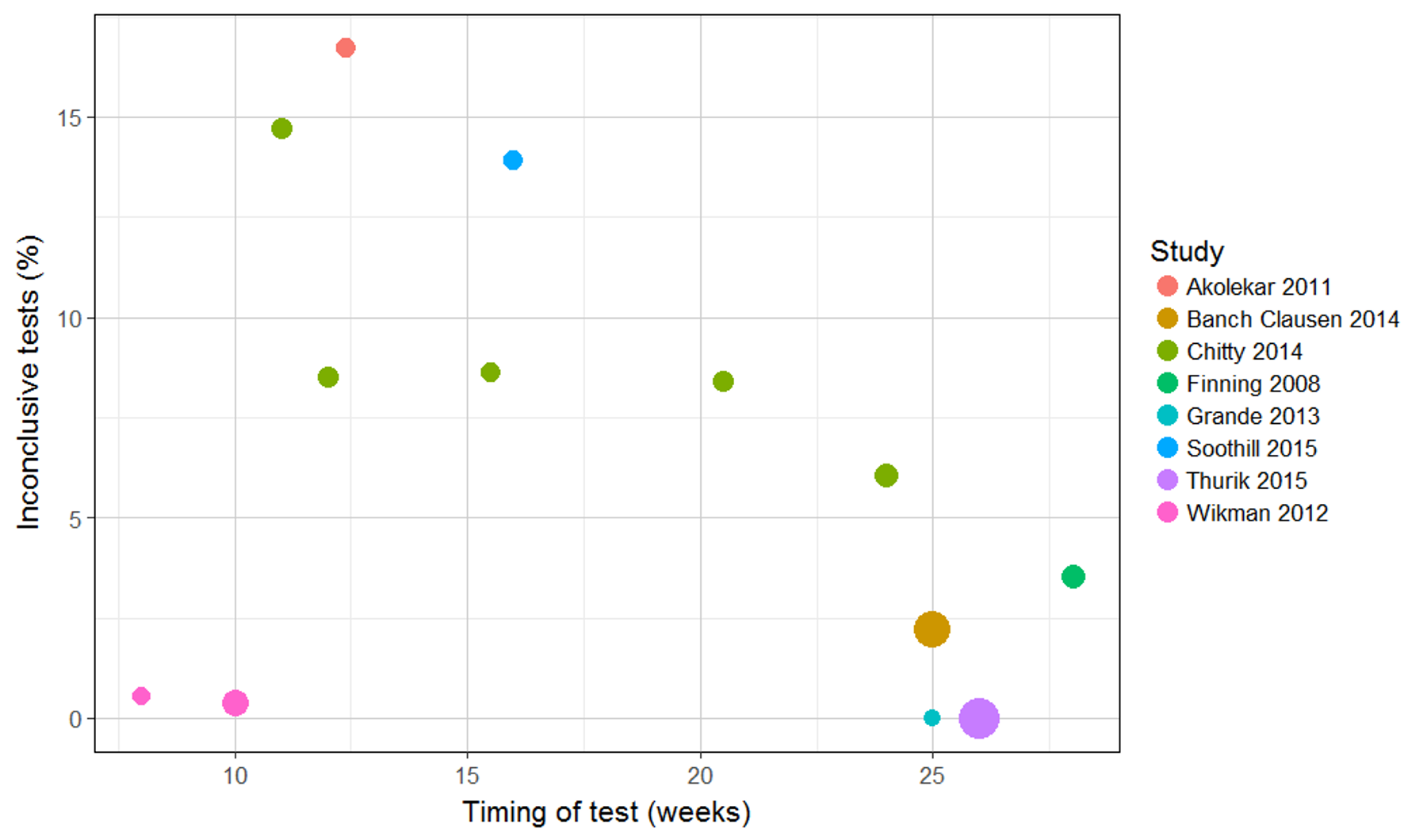

Supplement: Supplementary file 2 — Figure S1. False positive rate by gestational age at time of NIPT. (TIFF 241 kb) [file 12916_2019_1254_MOESM2_ESM.tiff]

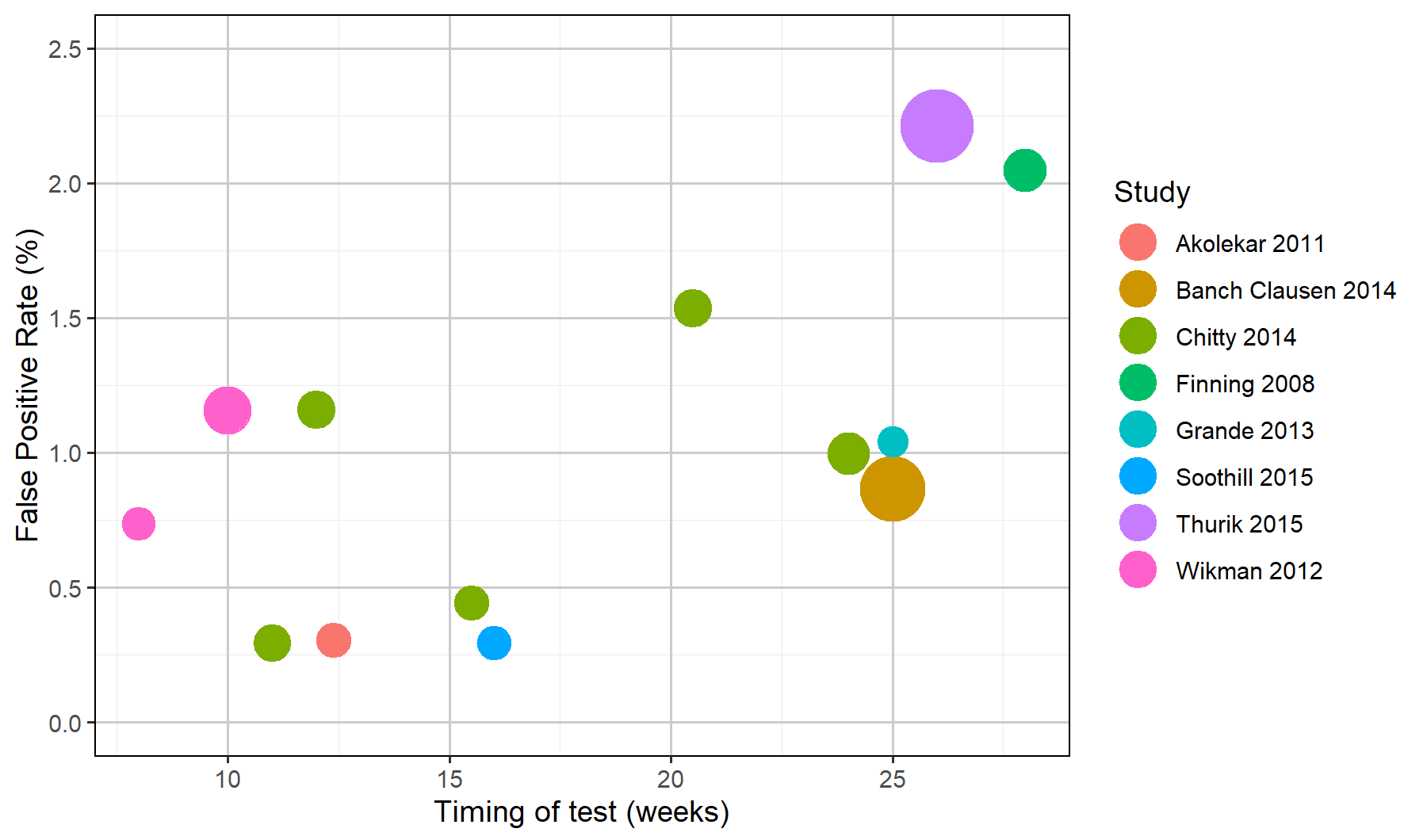

Supplement: Supplementary file 3 — Figure S2. Inconclusive results by test timing. (TIFF 67 kb) [file 12916_2019_1254_MOESM3_ESM.tiff]
