# Supplementary material for: High-throughput, non-invasive prenatal testing for fetal rhesus D status in RhD-negative women: a systematic review and meta-analysis
Source: BMC Med. 2019 Feb 14;17:37. doi: 10.1186/s12916-019-1254-4 (PMC6375191; doi:10.1186/s12916-019-1254-4)
Supplement: Supplementary file 4 — Probability estimates derived from published data, used in the simulation study. (DOCX 15 kb) [file 12916_2019_1254_MOESM4_ESM.docx]

Additional file 4: **Probability estimates derived from published data, used in the simulation study**

| **Probability** | **Estimate** | **Source** |
| --- | --- | --- |
|  |  |  |
| Rhesus positive fetus | 60.7% | Bristol-based diagnostic studies |
| Rhesus positive fetus (with inconclusive NIPT) | 70.7% | Bristol-based diagnostic studies |
| False negative NIPT test | 0.21% | Diagnostic meta-analysis (Bristol studies) |
| Inconclusive NIPT test | 6.7% | Bristol-based diagnostic studies |
| False-positive test (if conclusive) | 1.5% | Diagnostic meta-analysis (Bristol studies) * |
|  |  |  |
| Compliance with antenatal anti-D (without NIPT)  (received at least one dose of anti-D) | 99% | UK 2013 audit |
| Uptake of NIPT | 96% | De Haas 2012 (clinical effectiveness review) |
| Compliance with postpartum anti-D | 99% | UK 2013 audit |
| Compliance with antenatal anti-D  (if NIPT test refused or missed) | 80% | Soothill (2015) (clinical effectiveness review) |
| Compliance with antenatal anti-D  (if NIPT inconclusive) | 99% | Soothill (2015) |
| Uptake of antenatal anti-D in women with negative NIPT | 6% | Soothill (2015) |
| Compliance with postpartum anti-D after NIPT process | 99% | No data. Assumed same as without NIPT |
|  |  |  |
| Sensitisation with antenatal anti-D and post-partum anti-D | 0.35% | Pilgrim et al 2009 HTA report |
| Sensitisation with only postpartum anti-D | 0.95% | Pilgrim et al 2009 HTA report |
| Sensitisation with no anti-D | 10.7% | From Pilgrim, and Crowther et al. 1997 |
|  |  |  |
| Subsequent pregnancy in sensitised women | 62% | Used by Chitty (2014), no source given |
| Death of RhD negative fetus in sensitised women | 5% | Used by Chitty (2014), no source given |
|  |  |  |

** This result is based on a diagnostic meta-analysis of the Bristol-based studies, excluding women with inconclusive test results.*
